# Cognitive and affective Theory of Mind abilities in Parkinson’s disease before and 1 year after subthalamic deep brain stimulation

**DOI:** 10.3389/fnhum.2026.1699729

**Published:** 2026-03-23

**Authors:** Anna Elsenbach, Melanie Astalosch, Luísa Martins Ribeiro, Elias Landfried, Patricia Krause, Gerd-Helge Schneider, Gregor A. Brandt, Andrea A. Kühn, Dorothee Kübler-Weller

**Affiliations:** 1 Movement Disorder and Neuromodulation Unit, Department of Neurology and Experimental Neurology, Charité Universitätsmedizin Berlin, Freie Universität Berlin and Humboldt-Universität zu Berlin, Berlin, Germany; 2 Department of Neurosurgery, Charité Universitätsmedizin, Berlin, Germany; 3 Berlin School of Mind and Brain, Humboldt-Universität zu Berlin, Berlin, Germany; 4 Deutsches Zentrum für Neurodegenerative Erkrankungen, Berlin, Germany

**Keywords:** deep brain stimulation, mild cognitive impairment, Parkinson’s disease, social cognition, subthalamic nucleus, Theory of Mind

## Abstract

**Background:**

Parkinson’s disease (PD) is associated with specific cognitive deficits and dysfunctions in Theory of Mind (ToM), which can impact patients’ quality of life. ToM refers to the ability to infer the mental and emotional state of one’s counterpart and involves the prefrontal cortex and the basal ganglia in its function. While bilateral subthalamic nucleus deep brain stimulation (STN-DBS) in PD significantly improves motor symptoms, little is known about its effects on ToM.

**Methods:**

We prospectively applied an adapted version of the Yoni ToM paradigm, the Montréal Cognitive Assessment (MoCA), and an extensive tablet-based neurocognitive test set (CANTAB Connect™) before and 1 year after DBS surgery. Wilcoxon signed-rank tests were performed for comparisons of pre- and postoperative results, and Spearman’s correlations assessed the association between parameters with consequent Bonferroni corrections for multiple comparisons.

**Results:**

A total of 27 PD patients who received bilateral STN-DBS at Charité Universitätsmedizin Berlin between June 2019 and September 2021 were included in the study. Among these patients, 21 (77.8%) were men, with an average age of 58.7 ± 10.1 years and a disease duration averaging 10.2 ± 5.0 years. ToM abilities remained stable 1 year after DBS surgery and were associated with overall cognitive function and several specific neuropsychological domains, including reaction time, visual learning, and multitasking performance.

**Conclusion:**

Our findings suggest that specific ToM abilities and overall cognitive performance remain largely stable 1 year following STN-DBS. Performance on ToM tasks appears to be linked to individual cognitive profiles.

## 1 Introduction

Accumulating evidence indicates that Parkinson’s disease (PD) is associated with deficits in social cognition, particularly in Theory of Mind (ToM). ToM refers to the ability to infer other people’s mental states and feelings, which constitutes a pivotal pillar of social functioning in both humans and primates (Premack and Woodruff, 1978; McKinnon and Moscovitch, 2007). ToM can be subdivided into an affective subdomain (to infer others’ feelings) and a cognitive subdomain (to infer others’ thoughts) with different, but partially overlapping, neural mechanisms (Shamay-Tsoory and Aharon-Peretz, 2007; Shamay-Tsoory et al., 2007; Abu-Akel and Shamay-Tsoory, 2011). Research has already established key pathways in ToM processing—particularly the temporo-parietal junction, the precuneus, and the medial prefrontal cortex, along with higher functional connectivity networks (Carrington and Bailey, 2009; Rabini et al., 2024); however, the role of the basal ganglia in ToM remains elusive. In addition to their function in motor control, the basal ganglia and the subthalamic nucleus in particular appear to play important roles in ToM as well (Bodden et al., 2010; Bodden et al., 2013; Weintraub and Zaghloul, 2013).

Over the past two decades, growing evidence has suggested impairments in mentalizing abilities in PD patients: PD patients performed worse than matched controls in understanding social situations and mental states. However, these detectable ToM deficits did not necessarily reduce the quality of life (QoL) and were not associated with neuropsychiatric symptoms (Siripurapu et al., 2023). Meta-analytic data have revealed ToM impairments in PD patients in both verbal and visual tasks (Bora et al., 2015). Maggi et al. (2022a) have reported associations between affective ToM and lower education levels, reduced global cognition, and deficits in language and decision-making, whereas cognitive ToM deficits are related to older age, poorer executive function, and language impairments in PD patients. Still, there were no associations between ToM deficits and patients’ neuropsychiatric or motor symptom load as measured by traditional assessment scales (Maggi et al., 2022a). Whether ToM deficits in PD are linked to global cognitive decline or a dysexecutive syndrome often seen in PD patients with mild cognitive deficits (MCI) is currently a topic of debate (Maggi et al., 2022b). A recent study suggests that ToM impairment can occur early in PD, even in the absence of MCI, although it is associated with frontal executive deficits (Czernecki et al., 2021).

Bilateral subthalamic nucleus deep-brain stimulation (STN-DBS) is the effective treatment option for patients with advanced PD and significantly improves motor function and QoL (Tödt et al., 2022). While STN-DBS is generally deemed safe, postoperative global cognitive decline and minor deficits in executive functions, particularly verbal fluency, can occur in some patients after surgery (Witt, 2017; Hong et al., 2024; Bucur and Papagno, 2023; Tröster, 2024). The only longitudinally study investigating ToM in PD patients undergoing STN-DBS was conducted in 2010 and used 18F-FDG PET imaging. It found a significant decline in ToM abilities 3 months after surgery in comparison to 3 months before (*n* = 13). As ToM dysfunction is correlated with decreased cerebral glucose metabolism in several cortical areas, the authors concluded that STN-DBS impairs ToM abilities by modulating a widespread network (Péron et al., 2010a). On the other hand, Enrici et al. (2017) could not find a detrimental effect of STN-DBS on social cognition when comparing PD patients with sole medication and STN-DBS. Recently, Xiao et al. (2024) reported STN-DBS adversely affects cognitive ToM abilities, with this effect mediated by the associative STN, when comparing ToM abilities with DBS on versus off.

To recapitulate, ToM has been shown to be impaired in PD patients. The basal ganglia are involved in ToM networks, and their dysfunction can therefore be assumed to facilitate ToM deficits in PD. The role of the STN in ToM processing is not fully understood, but concerns have been raised about adverse effects of STN-DBS on mentalizing abilities in PD patients. As outlined, there are a few and partly contradictory results on this subject, and prospectively collected data have been lacking to date.

In this prospective cohort study, we investigated intra-individual ToM performance in patients with PD undergoing bilateral subthalamic DBS before surgery and 1 year after using an adapted Yoni paradigm alongside a comprehensive neuropsychological assessment to address two main research objectives: (1) How do ToM capacities evolve after DBS? and (2) To what extent are ToM capacities related to overall cognition and performance in specific neurocognitive domains?

## 2 Methods

### 2.1 Profiling of study cohort

This subset of ToM-specific analyses was conducted within a prospective cohort study registered at clinicaltrials.gov (NCT03982953) and approved by the institutional ethics committee (EA2/040/19). Study participants were included during their preoperative assessments at Charité Universitätsmedizin Berlin after having given their informed written consent. Inclusion criteria were a diagnosis of PD, an age >18 years, and a positive DBS evaluation, as well as sufficient German proficiency. The complete study setup can be found in the main paper (Kübler-Weller et al., 2025). Participants underwent an extensive test battery at two timepoints, preoperatively and 1 year after DBS surgery. Demographic parameters and medical information were surveyed in personal interviews. Motor symptom severity was ascertained through the Movement Disorder Society’s Unified Parkinson’s Disease Rating Scale (MDS-UPDRS) and QoL through the Parkinson’s Disease Questionnaire (PDQ-39). Questionnaires regarding comorbidities and non-motor PD symptoms were routinely assessed during pre- and postoperative stays and included the Activities of Daily Living Questionnaire (ADL), Beck Depression Inventory (BDI), Charlson Comorbidity Index (CKI), and the Starkstein Apathy Scale (SAS).

### 2.2 Neurocognitive testing

Participants completed the Montréal Cognitive Assessment (MoCA) as a cognitive screening test. Differential tablet-based cognitive testing was conducted with CANTAB Connect™. The test battery was customized for this study with a focus on assessing attention, visuospatial, as well as verbal memory, executive functions, and verbal association learning. A full description of all tasks can be found in the study cohort’s main paper (Kübler-Weller et al., 2025). Relevant to this publication were, inter alia, a visual pattern task (Pattern Recognition Task, PRT), the measurement of reaction time (Reaction Time Task, RTT), a memory task (Paired Associates Learning, PAL), and a multitasking trial with changing task cues (Multitasking Test, MTT). Additionally, ToM capacities were assessed using the Emotion Recognition Task (ERT), where emotional states have to be inferred from facial expressions. The evaluated parameters were specific to each task and included reaction time, correct answers, error rates, or calculated strategy points.

**Figure 1:**
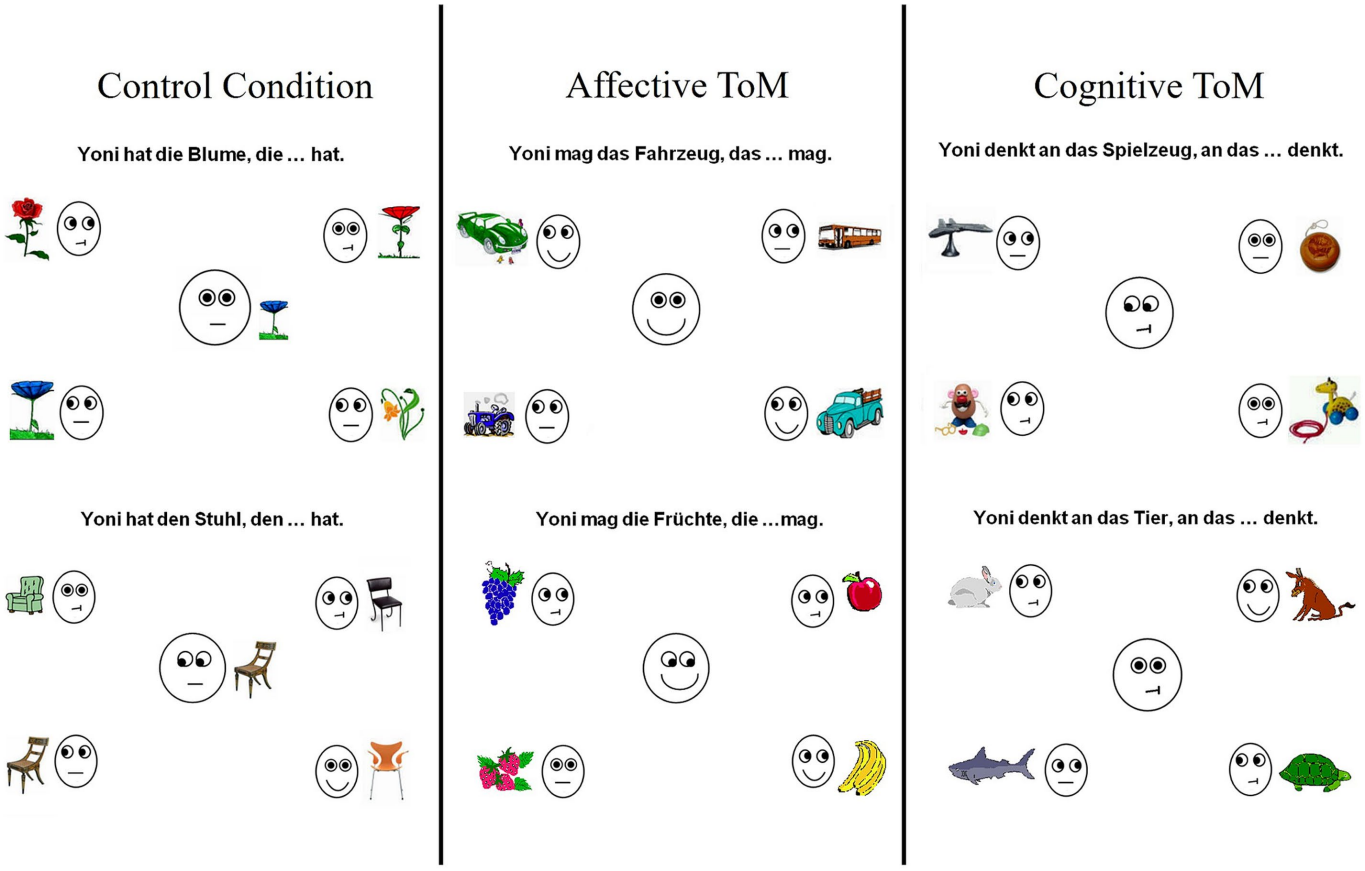
Exemplary test items of the adapted version of the Yoni paradigm contain an affective and cognitive ToM condition as well as a control condition with 20 items each. Gaze directions are balanced across conditions.

In order to further investigate ToM, an adapted version (Bodden et al., 2013) of the well-established Yoni paradigm (Shamay-Tsoory and Aharon-Peretz, 2007) testing only second-order ToM was used. It is a computed visual task centered around a character named Yoni and contains an affective, cognitive, and control second-order ToM condition. Examples are depicted in Figure 1. Yoni is located in the center of the screen with distinct facial expressions and directions of gaze. It is surrounded by objects and paired with written text cues asking to infer either its cognitive (“What is Yoni thinking about?”) or affective mental state (“Which item does Yoni like?”). Drawing on the written information together with the mouth shape and gaze directions, test subjects must thus infer the thoughts or emotions of the character “Yoni” and answer the questions. The control condition requires participants to identify matching objects and omits mental state inferences. Outcome parameters of the paradigm are accuracy (percentage of correctly answered trials) and response times.

All tests were conducted with patients on optimized medication (Med ON) and stimulation (DBS ON).

### 2.3 Statistical analyses

Statistical analyses were conducted using IBM SPSS Statistics Version 30. Descriptive statistics include mean and standard deviation (SD). As the data were not normally distributed according to Kolmogorov–Smirnov testing, Wilcoxon signed-rank tests were used for comparisons of pre- and postoperative results, and *Z* scores were computed to evaluate the significance of calculated differences. Spearman’s correlations assessed associations between ToM abilities and non-motor as well as other cognitive characteristics. Due to the multitude of comparisons, it was decided to use a conservative Bonferroni correction of the *α* level (*p* < 0.05), resulting in a new significance level of *p*_corr_ < 0.001. Graphs were plotted using GraphPad Prism Version 10.

## 3 Results

### 3.1 Evolution of motor and non-motor symptoms after STN-DBS

A total of 27 PD patients who received bilateral STN-DBS at Charité Universitätsmedizin Berlin between June 2019 and September 2021 completed the Yoni paradigm prior to and 1 year after STN-DBS surgery (1yFU) and were therefore included in this analysis (21 male (77.8%), age 58.7 ± 10.1 years, disease duration 10.2 ± 5.0 years). Comprehensive cohort characteristics are presented in Table 1.

**Table 1:** Descriptive data from the ToM subcohort with pre- and postoperative results from all the surveyed questionnaires and neurocognitive tests.

	*n*	Preoperative mean ± SD	Postoperative mean ± SD	*Z* score	*p*
**Comorbidity questionnaires**					
Activities of daily living questionnaire	22	14.1 ± 11.2	10.7 ± 12.3	−1.852	0.064
Beck depression inventory	23	11.5 ± 7.1	10.7 ± 7.6	−0.488	0.626
Charlson comorbidity index	27	0.5 ± 0.8	0.4 ± 0.6	−1.265	0.206
Starkstein apathy scale	20	15.6 ± 6.2	13.9 ± 6.1	−0.947	0.344
**PD-specific parameters**					
Levodopa equivalent daily dose	27	1,141.2 ± 325.9	588.9 ± 366.2	−4.381	<0.001
MDS-UPDRS I	21	9.2 ± 4.7	7.4 ± 4.7	−1.203	0.229
MDS-UPDRS II	22	10.7 ± 5.9	9.4 ± 7.6	−1.335	0.182
MDS-UPDRS III ON (preoperative: medication, postoperative: medication and DBS)	18	24.2 ± 8.3	18.6 ± 9.6	−2.253	0.024
MDS-UPDRS IV	17	5.8 ± 5.5	2.2 ± 2.4	−2.247	0.025
Parkinson’s Disease Questionnaire (PDQ39)					
PDQ39: mobility (in percent)	21	29.9 ± 20.2	12 ± 8.8	−3.530	<0.001
PDQ39: daily activities (in percent)	21	30.9 ± 19.3	5.4 ± 4.7	−3.733	<0.001
PDQ39: emotional wellbeing (in percent)	21	28.4 ± 17.5	5 ± 4.6	−3.921	<0.001
PDQ39: stigma (in percent)	21	21.8 ± 23.4	1.8 ± 2.4	−3.528	<0.001
PDQ39: social support (in percent)	21	13.9 ± 17.4	1.64 ± 2.5	−3.125	0.002
PDQ39: cognition (in percent)	21	30 ± 19.9	4.9 ± 3.8	−3.808	<0.001
PDQ39: communication (in percent)	21	19.2 ± 16.9	3.3 ± 2.6	−3.664	<0.001
PDQ39: bodily discomfort (in percent)	21	33.8 ± 28.2	3.1 ± 2.5	−3.824	<0.001
Parkinson’s Disease Sum Index (PDSI)	21	32.6 ± 10.2	4.6 ± 2.8	−3.621	<0.001
**Cognitive testing**					
Montréal Cognitive Assessment (MoCA)	27	25.0 ± 2.9	25.4 ± 2.8	−0.370	0.711
Yoni ToM task					
Cognitive ToM accuracy (in percent)	27	79.7 ± 18.5	78.0 ± 17.9	−1.010	0.313
Cognitive ToM response time (in s)	27	5.3 ± 4.3	5.6 ± 4.9	−0.580	0.556
Affective ToM accuracy (in percent)	27	76.1 ± 22.4	77.6 ± 22.2	−0.183	0.855
Affective ToM response time (in s)	27	5.5 ± 4.9	6.7 ± 4.7	−1.514	0.130
Physical control accuracy (in percent)	27	76.04 ± 21.7	78.2 ± 14.4	−0.057	0.954
Physical control response time (in s)	27	4.7 ± 3.5	7.1 ± 5.2	−2.102	0.036
CANTAB Connect™ test battery					
MOTML—motor screening test mean latency (in ms)	27	1,006.6 ± 413.7	1,060.4 ± 254.8	−1.201	0.230
RTISMRT—reaction time task simple median reaction time (in ms)	27	374.6 ± 65.8	374.4 ± 75.1	−0.180	0.857
RTIFMDRT—reaction time task median five-choice reaction time (in ms)	27	416.0 ± 59.6	440.3 ± 80.6	−1.740	0.082
PRMPCI—pattern recognition memory correct immediate	26	84.6 ± 9.9	79.7 ± 13.9	−1.772	0.076
SSPFSL—spatial span forward span length	27	5.3 ± 1.4	5.4 ± 1.0	−0.251	0.802
SSPRSL—spatial span reverse span length	27	5.2 ± 1.2	5.1 ± 1.2	−0.346	0.729
ERTTH—emotion recognition task total hits	27	24.6 ± 4.5	22.5 ± 6.1	−2.29	0.022
PRMPCD—pattern recognition memory percent correct delayed	26	72.9 ± 14.3	71.6 ± 17.2	−0.179	0.858
VRMFRDS—verbal recognition memory free recall: distinct stimuli	27	3.5 ± 2.3	3.9 ± 1.9	−1.150	0.252
VRMIRTC—verbal recognition memory immediate recognition	27	28.3 ± 3.0	27.4 ± 4.5	−0.752	0.452
MTTTIC—multitasking test total incorrect	27	18.4 ± 20.7	17.0 ± 16.1	−0.445	0.656
MTTLMD—multitasking test reaction latency (median, in ms)	27	806.7 ± 127.2	849. ± 157.8	−1.270	0.203
MTTICMD—multitasking test incongruency cost (median, in ms)	27	83.6 ± 69.8	77.3 ± 79.5	−0.495	0.620
MTTMTCMD—multitasking test multitasking cost (median, in ms)	27	313.9 ± 172.5	259.6 ± 167.4	−0.985	0.325
SWMBE—spatial working memory between errors	27	17.9 ± 7.3	17.3 ± 8.8	−0.256	0.798
SWMS—spatial working memory strategy (6–8 boxes)	27	8.3 ± 3.8	5.7 ± 4.8	−2.940	0.003
PALTEA—paired associates learning total errors (adjusted)	27	24.7 ± 16.2	25.3 ± 20.2	−0.132	0.895
PALFAMS—paired associates learning first attempt memory score	27	8.8 ± 4.0	9.4 ± 4.8	−0.662	0.508

Results are given as mean ± standard deviation (SD). Wilcoxon signed-rank tests were used to compare pre- and postoperative results. *Z* scores were calculated using rank sums, including the distribution, and indicate the magnitude of the difference.

STN-DBS resulted in a marked reduction in dopaminergic medication requirements (*Z* = −4.381, *p* < 0.001). Motor symptom severity improved as evidenced by reductions in MDS-UPDRS Part III (*Z* = −2.253, *p* = 0.024) and Part IV scores (*Z* = −2.247, *p* = 0.025). Patients reported improvements in their QoL with an overall significant reduction in PDQ-39 Summary Index (PDSI) (*Z* = −3.621, *p* < 0.001). No significant postoperative changes were observed in the Activities of Daily Living Questionnaire (*Z* = −1.852, *p* = 0.064), Beck Depression Inventory (*Z* = −0.488, *p* = 0.626), Charlson Comorbidity Index (*Z* = −1.265, *p* = 0.206), or Starkstein Apathy Scale (*Z* = −0.947, *p* = 0.344).

### 3.2 Neurocognitive profiles before and after STN-DBS

**Figure 2:**
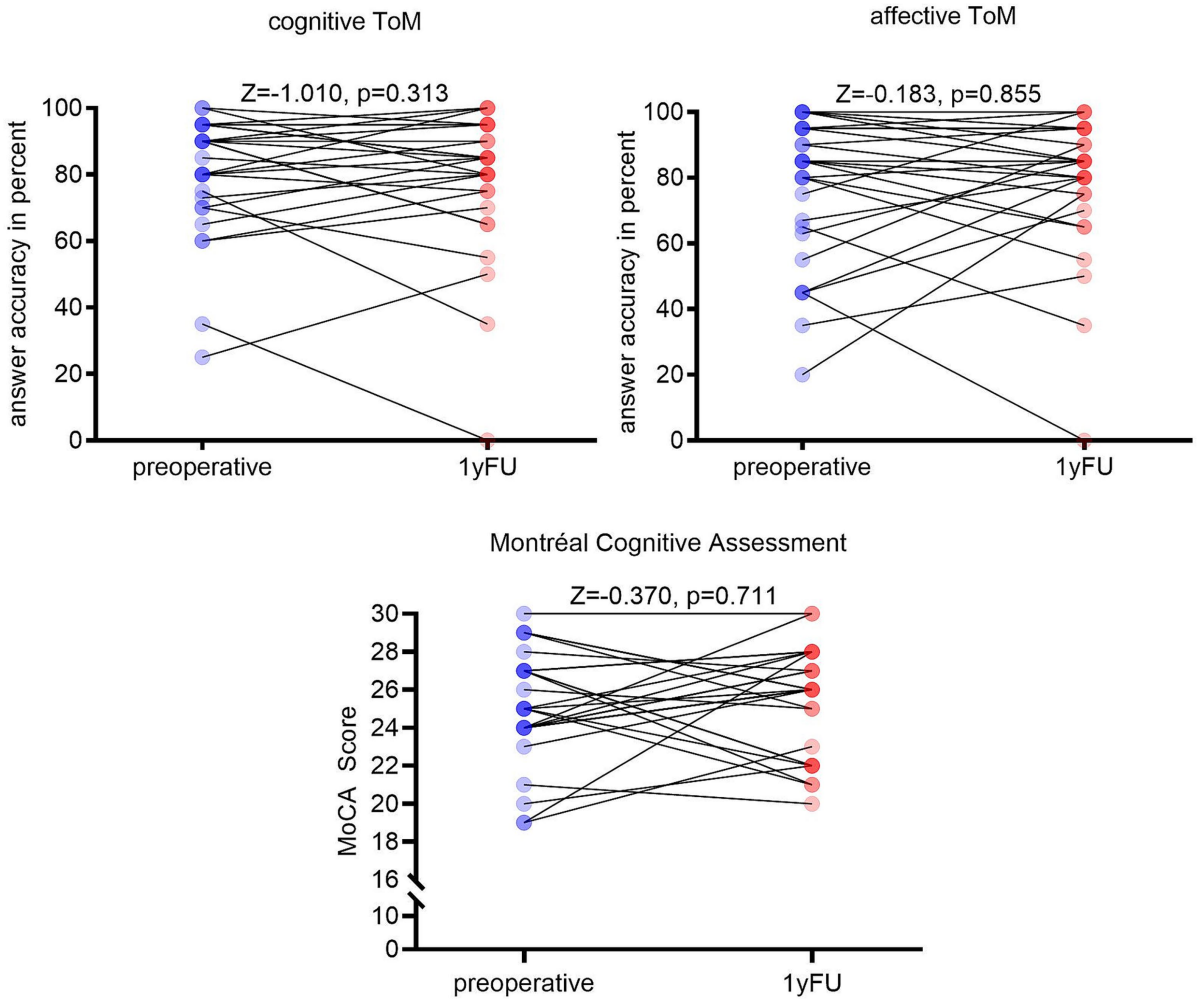
Comparison of cognitive and affective ToM accuracy as well as Montréal Cognitive Assessment (MoCA) score. Answer accuracy is given in percent, and MoCA performance is given in numerical scores. Blue indicates pre- and red postoperative results. No significant changes are observed.

No significant change in overall cognitive function as measured by the MoCA was observed (*Z* = −0.370, *p* = 0.711). However, preoperative cognitive screening indicated minor cognitive deficits in most patients, fulfilling the MoCA-based MCI criteria in 17/27 patients (Nasreddine et al., 2005). Concerning ToM results, neither affective nor cognitive ToM performance changed significantly 1 year postoperatively in comparison to baseline. Both accuracy (cognitive ToM: *Z* = −1.010, *p* = 0.313; affective ToM: *Z* = −0.183, *p* = 0.855) and response time (cognitive ToM: *Z* = −0.580, *p* = 0.556; affective ToM: *Z* = −1.514, *p* = 0.130) stayed stable within the study cohort (see Figure 2). Overall, we observed slower response times under all conditions without reaching significance. In the differential neuropsychology test set conducted with CANTAB Connect™, there were no significant differences in performance pre- versus 1 year postoperatively, either.

### 3.3 Correlations between ToM abilities and neurocognitive measures

**Figure 3:**
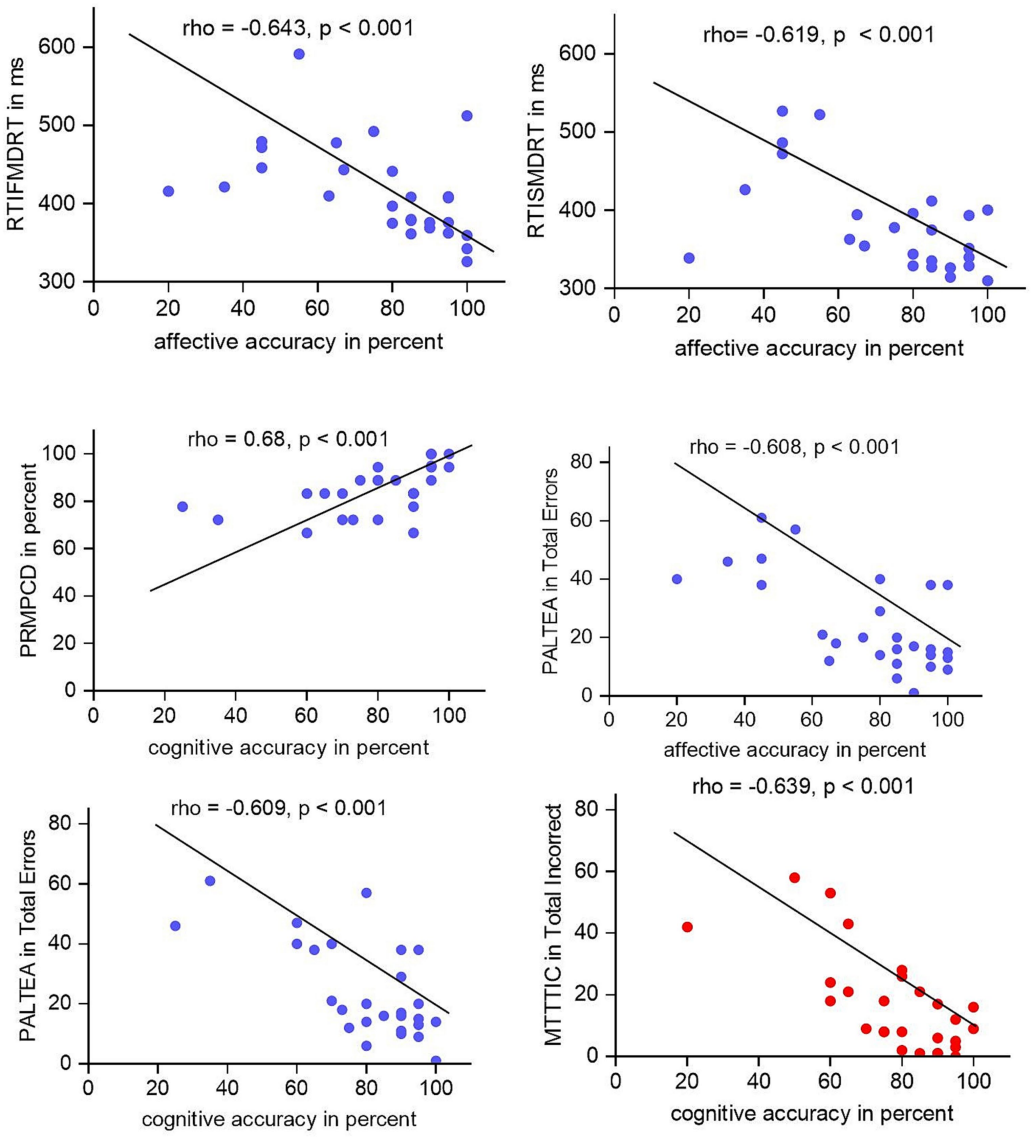
Significant pre- and postoperative correlations between CANTAB Connect™ measures and ToM conditions: RTIFMDRT in ms, RTISMDRT in ms, PRMPCD in percent, PALTEA in total errors, and MTTTIC in total incorrect. ToM accuracy is given in percentage. Preoperative data are given in blue, postoperative in red. RTISMRT, Reaction Time Task Simple Median Reaction Time; RTIFMDRT, Reaction Time Task Median Five-Choice Reaction Time; PRM, Pattern Recognition Memory; PAL, Paired Associates Learning; MTT, Multitasking Test.

To assess associations between ToM abilities and other neurocognitive as well as clinical parameters, Spearman correlations were applied. Due to the extent of comparisons, a Bonferroni-corrected *p* < 0.001 was deemed significant. Postoperative cognitive ToM accuracy correlated significantly with MoCA results (rho = 0.653, *p* < 0.001), with a similar tendency for the affective condition (rho = 0.542, *p* = 0.003). This correlation was not found for the Yoni control condition (rho = 0.293, *p* = 0.138). Regarding CANTAB Connect™ performance, we found associations between ToM and several items. Preoperatively, the following results reached the Bonferroni-corrected significance level: Reaction Time Task Simple Median Reaction Time and Reaction Time Task Median Five-Choice Reaction Time both correlated negatively with affective ToM accuracy (Median Reaction Time: rho = −0.619, *p* < 0.001; Five-Choice Reaction Time: rho = −0.643, *p* < 0.001). Furthermore, the number of correct answers in the Pattern Recognition Task correlated positively with cognitive ToM accuracy (rho = 0.68, *p* < 0.001). Additionally, the number of errors in the Paired Associates Learning Task showed a negative correlation with accuracy in the affective (rho = −0.61, *p* < 0.001) and cognitive conditions (rho = −0.61, *p* < 0.001). In the postoperative data set, the number of incorrect answers in the multitasking test correlated negatively with cognitive ToM accuracy (rho = −0.639, *p* < 0.001). Significant correlations are shown as scatterplots in Figure 3. Statistical data are supplied in Supplementary material.

## 4 Discussion

This is, to our knowledge, the first study prospectively assessing ToM in the context of bilateral STN-DBS in PD patients. Overall, ToM abilities were stable 1 year after DBS surgery and associated with global cognition and a number of specific neuropsychological domains.

In the Yoni task, neither affective nor cognitive ToM accuracy or response times changed significantly 1 year postoperatively compared to baseline, despite marked improvement in motor symptoms. Patients also showed no statistically significant decline in emotion recognition capacity determined by the ERT from the CANTAB Connect™ Test battery. Our results suggest that STN-DBS does not systematically alter ToM abilities at the group level, in line with findings by Enrici et al. (2017). Our data also support more recent reports of sustained emotion recognition post-DBS (Duits et al., 2024) and apply to the entire study cohort as well (Kübler-Weller et al., 2025). This stability, however, contradicts older reports of negative DBS effects on social cognition, namely emotion recognition as reported by Péron et al. (2010a), Péron et al. (2010b), and Dujardin et al. (2004). They found a significant decline in the Reading the Mind in the Eyes task 3 months after surgery compared to preoperatively, with no correlation to overall cognition. ToM deficits were associated with cerebral hypometabolism, thus leading to the conclusion of a negative STN-DBS effect on the ToM network. However, due to the complex study setup, they only included 13 patients. Furthermore, their primary testing point was 3 months after electrode implantation, which is generally considered quite early the recovery time, as definitive programming and medication adjustments might still be ongoing. As the authors discussed back then, a temporary microlesion effect of STN-DBS could also explain the impaired emotion recognition 3 months after surgery. In contrast, we therefore chose to test patients 1 year after surgery. Our findings suggest the stability of abilities, as we could not find any statistically significant impairment after 1 year. Duits et al. (2024)’s recent paper reports no detrimental effect of STN-DBS on emotion recognition 1 year after surgery.

Better performance in the cognitive ToM condition was associated with better overall cognition as measured by the MoCA 1 year after DBS surgery. This correlation speaks in favor of the proposed link between ToM and overall cognitive abilities (Bora et al., 2015; Maggi et al., 2022a; Maggi et al., 2022b). In contrast, Czernecki et al. could not corroborate this association and postulated an independent ToM impairment: They found significant deficits in social cognition in their cohort independent of MCI diagnoses and thus not mediated by global cognitive deficits (Czernecki et al., 2021). Menozzi et al. (2025) even claim that affective ToM deficits can be classified as a “non-motor feature of PD” as their study cohort also lacked a connection between ToM and overall cognition. Our results, however, are in line with the majority of ToM studies and indicate a correlation between overall cognition and ToM abilities in PD patients with STN-DBS. Parts of the discrepant results may be explained by the different disease stages of the examined patients and ToM paradigms used: Czernecki et al. used the Faux Pas Test to examine ToM, which is more complex and relies heavily on the conductor’s assessment. They noted that patients often attributed false intentionality and seemed blunter in answering, which conversely could also be attributed to neuropsychiatric facets of PD, such as impulsivity. Their cohort was demographically quite similar to ours (except for treatment, in our case, DBS). Menozzi et al. used similar ToM tasks, e.g., the Reading the Mind in the Eyes test (which is comparable to the ERT in our CANTAB Connect™ test), but their patients were much older (mean of 70.65 years) and less educated (mean of 9.15 years) than ours. These discrepancies illustrate the need for prospective studies with larger and more diverse cohorts in order to further understand the interactions between cognition, ToM, and PD as well as the role of STN.

Concerning the differential neuropsychological test set conducted by means of CANTAB Connect™, a number of measures corresponded to ToM performance: Preoperatively, higher cognitive and affective ToM accuracy was associated with faster reaction times and better visual association learning. Higher cognitive ToM accuracy was additionally associated with superior pattern recognition skills. Postoperatively, cognitive ToM accuracy correlated with global cognition as measured by the MoCA and multitasking performance (fewer errors). Notably, correlations between performance in ToM and other specific neuropsychological domains such as reaction time, pattern recognition, and association learning indicate that both speed of information processing and associative memory contribute to ToM performance, both in the affective and cognitive domains. In line with our findings, Trompeta et al. (2021) underline correlations between ToM abilities and executive functions, language, and visuospatial memory. The strong association between cognitive ToM accuracy and multitasking ability postoperatively further emphasizes the association between executive control and mental state inference.

Furthermore, we can report an overall drastic effect on QoL, measured in sum by the PDSI 1 year after DBS. Ni et al. (2025) recently came to similar conclusions retrospectively analyzing QoL and cognitive decline in DBS patients. Overall QoL was reported as significantly higher post-DBS surgery, even for already cognitively impaired patients. We hence assume that the impact of decreased PD symptoms with an improvement in mobility, independence, and social participation has an immense effect on subjective QoL ratings and might outweigh possible mild cognitive and ToM deficits. Supporting this, Siripurapu et al. (2023) in turn have discussed that in their PD cohort, overall QoL was not equally affected by measurable impaired social cognition.

Several limitations of this study warrant consideration. The sample size was modest, potentially underpowering the detection of small DBS-related effects. In order to increase the statistical power, we decided to correct for multiple testing, resulting in a cautious *α* level. This therefore reduced the number of reported significances, although we consider the reported results especially robust. Moreover, the focus on second-order ToM using a simplified paradigm may not capture the full breadth of social cognitive changes. Furthermore, all ToM tasks depend heavily on motor performance and overall cognitive abilities, which might skew results as PD patients often display independent deficits in these areas. As the MoCA is a screening tool, its limitations in detecting subtle or domain-specific changes must additionally be taken into consideration.

Furthermore, we did not recruit either a healthy or a disease-matched non-DBS control group, which limits the conclusions, as it does not factor in natural disease progression as well as DBS effects and, in turn, lays a focus on intra-individual change.

Analyses of electrode locations and STIM-ON/OFF protocols were omitted due to the focus on long-term intraindividual ToM development; they are, however, an important aspect of our continuous efforts to understand cognition within PD as well as in STN-DBS patients.

Future studies should integrate multimodal ToM assessments, stimulation analyses, and longitudinal follow-up beyond 1 year to better delineate ToM evolvement in PD after STN-DBS.

In conclusion, our findings suggest that while STN-DBS improves motor symptoms and overall QoL in PD, global cognition and specific ToM abilities 1 year after surgery only change minimally at the group level and are tied to individual cognitive profiles. This underscores the need for comprehensive neuropsychological evaluation of DBS candidates and highlights the cognitive-social interplay as a potential target for adjunctive rehabilitation strategies.

## Data Availability

The raw data supporting the conclusions of this article will be made available by the authors without undue reservation.
