# Supplementary material for: Cognitive and affective Theory of Mind abilities in Parkinson’s disease before and 1 year after subthalamic deep brain stimulation
Source: Front Hum Neurosci. 2026 Mar 23;20:1699729. doi: 10.3389/fnhum.2026.1699729 (PMC13050947; doi:10.3389/fnhum.2026.1699729)
Supplement: Supplementary file 1 [file Supplementary_file_1.pdf]

Significant ($p < 0.05$) Results of Spearman Rho Correlations, Bonferroni Corrected Significancies printed in bold:

**preoperative parameters**	**ToM Yoni Task Item**	**n**	**rho**	**p**
age	cognitive response time	21	0.548	0.01
age	physical response time	21	0.545	0.011
CANTAB Connect Test Battery				
Pattern Recognition Memory Correct Immediate	cognitive accuracy	27	0.68	**<.001**
Pattern Recognition Memory Correct Immediate	affective accuracy	27	0.593	**0.001**
Pattern Recognition Memory Correct Immediate	physical accuracy	27	0.585	**0.001**
Spatial Span Forward Span Length	cognitive accuracy	27	0.487	0.01
Spatial Span Forward Span Length	affective accuracy	27	0.46	0.016
Spatial Span Forward Span Length	physical accuracy	27	0.448	0.019
Multitasking Test Total Incorrect	cognitive accuracy	27	-0.442	0.021
Multitasking Test Total Incorrect	affective accuracy	27	-0.495	0.009
Multitasking Test Total Incorrect	physical accuracy	27	-0.482	0.011
Multitasking Test Reaction Latency (Median)	cognitive accuracy	27	-0.413	0.032
Multitasking Test Reaction Latency (Median)	physical accuracy	27	-0.508	0.007
Multitasking Test Multitasking Cost (Median)	physical response time	27	0.408	0.035
Paired Associates Learning Total Errors (Adjusted)	cognitive accuracy	27	-0.609	**<.001**
Paired Associates Learning Total Errors (Adjusted)	affective accuracy	27	-0.608	**<.001**
Paired Associates Learning Total Errors (Adjusted)	physical accuracy	27	-0.504	0.007
Paired Associates Learning First Attempt Memory Score	cognitive accuracy	27	0.48	0.011
Paired Associates Learning First Attempt Memory Score	affective accuracy	27	0.48	0.011
Paired Associates Learning First Attempt Memory Score	physical accuracy	27	0.388	0.046
**postoperative parameters**	**ToM**	**n**	**rho**	**p**
CKI	cognitive accuracy	27	-0.499	0.008
CKI	affective response time	27	0.423	0.028
CKI	affective accuracy	27	-0.477	0.012
LEDD	cognitive response time	27	-0.489	0.01
LEDD	affective response time	27	-0.406	0.036

UPDRS_I	cognitive accuracy	22	-0.613	0.002
UPDRS_II	physcial response time	23	0.463	0.026
UPDRS_III_S-OFF_M-ON	cognitive response time	23	0.577	0.004
UPDRS_IV	cognitive response time	19	-0.600	0.007
years of education	affective accuracy	27	0.423	0.028
MOCA	cognitive accuracy	27	0.653	**<0.001**
MOCA	affective accuracy	27	0.542	0.003
BDI	physical accuracy	24	-0.389	0.06
BDI	cognitive accuracy	24	-0.514	0.01
MoCA difference	cognitive accuracy change	27	0.402	0.037
Parkinson's Disease Questionnaire (39 Questions)				
PDQ39:Mobility	cognitive accuracy	22	-0.567	0.006
PDQ39:Mobility	affective response time	22	0.58	0.005
PDQ39:Cognition	cognitive accuracy	22	-0.505	0.017
PDQ39:Communication	cognitive accuracy	22	-0.455	0.034
PDQ39:Bodily Discomfort	cognitive accuracy	22	-0.574	0.005
PDQ39: Emotional Well-Being	cognitive accuracy	22	-0.492	0.02
PDQ39:Daily Activities	affective response time	22	0.548	0.008
PDQ39:Daily Activities	cognitive response time	22	0.493	0.02
PDQ39:Daily Activities	cognitive accuracy	22	-0.465	0.029
CANTAB Connect Test Battery				
Reaction Time Task Simple Median Reaction Time	cognitive response time	27	0.401	0.038
Reaction Time Task Median Five-Choice Reaction Time	cognitive response time	27	0.399	0.039
Reaction Time Task Median Five-Choice Reaction Time	affective response time	27	0.479	0.011
Pattern Recognition Memory Correct Immediate	cognitive accuracy	26	0.425	0.03
Pattern Recognition Memory Correct Immediate	affective accuracy	26	0.577	0.002
Pattern Recognition Memory Correct Immediate	physical accuracy	26	0.445	0.023
Spatial Span Forward Span Length	cognitive accuracy	27	0.39	0.044
Spatial Span Reverse Span Length	cognitive accuracy	27	0.424	0.028

Spatial Span Reverse Span Length	affective accuracy	27	0.547	0.003
Spatial Span Reverse Span Length	physical accuracy	27	0.468	0.014
Verbal Recognition Memory Free Recall: Distinct Stimuli	cognitive accuracy	27	0.41	0.034
Verbal Recognition Memory Free Recall: Distinct Stimuli	physical accuracy	27	0.403	0.037
Multitasking Test Total Incorrect	cognitive accuracy	27	-0.639	**<.001**
Multitasking Test Total Incorrect	affective accuracy	27	-0.55	0.003
Multitasking Test Total Incorrect	physical accuracy	27	-0.389	0.045
Multitasking Test Reaction Latency (Median)	cognitive accuracy	27	0.,425	0.027
Spatial Working Memory Between Errors	cognitive accuracy	27	-0.477	0.012
Spatial Working Memory Strategy (6-8 Boxes)	cognitive response time	27	0.596	**0.001**
Spatial Working Memory Strategy (6-8 Boxes)	affective response time	27	0.567	0.002
Paired Associates Learning Total Errors (Adjusted)	cognitive accuracy	27	-0.415	0.032
Verbal Recognition Memory Delayed Recognition	physical response time	27	0.401	0.047
Emotion Recognition Task Total Hits	PDQ39:Daily Activities	22	-0.443	0.039
